# *Vibrio fluvialis* in Patients with Diarrhea, Kolkata, India

**DOI:** 10.3201/eid1811.120520

**Published:** 2012-11

**Authors:** Goutam Chowdhury, Gururaja P. Pazhani, Devarati Dutta, Sucharita Guin, Sanjucta Dutta, Santanu Ghosh, Hidemasa Izumiya, Masahiro Asakura, Shinji Yamasaki, Yoshifumi Takeda, Eiji Arakawa, Haruo Watanabe, Asish K. Mukhopadhyay, Mihir K. Bhattacharya, K. Rajendran, Gopinath Balakrish Nair, Thandavarayan Ramamurthy

**Affiliations:** National Institute of Cholera and Enteric Diseases, Kolkata, India (G. Chowdhury, G.P. Pazhani, D. Dutta, S. Guin, S. Dutta, S. Ghosh, A.K. Mukhopadhyay, M.K. Bhattacharya, K. Rajendran, G. Nair, T. Ramamurthy);; National Institute of Infectious Diseases, Tokyo, Japan (H. Izumiya, E. Arakawa, H. Watanabe);; Osaka Prefecture University Graduate School of Life and Environmental Sciences, Osaka, Japan (M. Asakura, S. Yamasaki);; and National Institute of Cholera and Enteric Diseases Collaborative Research Center of Okayama University for Infectious Diseases in India, Kolkata (Y. Takeda).

**Keywords:** Diarrhea, V. fluvialis, Vibrio fluvialis, Vibrio, antimicrobial resistance, PFGE, pulsed-field gel electrophoresis, enteric infections, bacteria, India

## Abstract

We identified 131 strains of *Vibrio fluvialis* among 400 nonagglutinating *Vibrio* spp. isolated from patients with diarrhea in Kolkata, India. For 43 patients, *V. fluvialis* was the sole pathogen identified. Most strains harbored genes encoding hemolysin and metalloprotease; this finding may contribute to understanding of the pathogenicity of *V. fluvialis*.

Many members of the family *Vibrionaceae* cause diarrheal disease; among these, *Vibrio cholerae* O1/O139 and *V. parahaemolyticus* are responsible for several epidemics and pandemics (*1*,*2*). In Indonesia, >20% of diarrheal infections are caused by pathogenic *Vibrio* spp (*3*). Some of these *Vibrio* spp. can grow in thiosulfate–citrate–bile salts–sucrose agar as yellow colonies and do not agglutinate with *V. cholerae* O1 antiserum. These species are broadly defined as nonagglutinating (NAG) vibrios.

The emerging etiologic agent *V. fluvialis* has caused sporadic cases and outbreaks of diarrhea in several countries (*4*–*6*). Species-specific minimal biochemical tests, e.g., lysine decarboxylase, ornithine decarboxylase, arginine didydrolase, and L-arabinose, are used to identify *V. fluvialis*; without these tests, it may be confused with NAG vibrios, *V. cholerae*, and even *Aeromonas* spp. In most resource-poor countries, these tests are not performed, which may lead to labeling of *V. fluvialis* as a NAG vibrio.

Although *V. fluvialis* is known to cause diarrhea, the mechanisms involved in its pathogenicity are not well established. To evaluate the prevalence of *V. fluvialis* in India and possible mischaracterization as an NAG vibrio, we examined cases in which isolates from hospitalized patients with diarrhea were identified as NAG vibrios and characterized the strains using phenotypic and genetic methods.

## The Study

We examined 400 isolates identified as NAG vibrios that were collected during 2002–2009 from 11,904 stool specimens from patients with diarrhea admitted to the Infectious Diseases and Beliaghata General Hospital, Kolkata, India. Specimens were screened for common enteric pathogens, according to standard protocols (*7*). Oxidase, string test, and arginine dihydrolase–positive strains that did not agglutinate with *V. cholerae* O1 polyvalent or O139 monovalent antiserum were further confirmed as *V. fluvialis* by using a multiplex PCR targeting the *toxR* gene of *V. fluvialis* and the *ompW* gene of *V. cholerae* (*8*,*9*). Isolates were also subjected to PCRs targeting different virulence-associated genes encoding the repeat in toxin (*rtxA, rtxC*), heat-stable enterotoxin (*stn*), type 3 secretion system (*vcsC2, vcsV2, vcsN2* and *vspD*), cholera toxin (*ctxA*), toxin co-regulated pilus (*tcpA*), thermostable direct-hemolysin (*tdh*), TDH-related hemolysin (*trh*), *V. fluvialis* hemolysin (VFH), and metalloproteases, according to published methods (*10*–*12*).

Expression of VFH was determined in vitro by using erythrocytes from rabbit and sheep. Cytotoxin assay was performed with HeLa and Chinese hamster ovary cell lines by using sterile culture filters of the *V. fluvialis* strains that were isolated as a sole pathogen. Antimicrobial drug susceptibility testing was performed by using the disk diffusion method with commercially available disks (Becton Dickinson, Sparks Glencoe, MD, USA), according to Clinical and Laboratory Standards Institute criteria (*13*). Because these guidelines do not include interpretive criteria for *V. fluvialis*, breakpoints for *Enterobacteriaceae* were adopted. *Escherichia coli* ATCC 25922 was used as a quality control strain.

Pulsed-field gel electrophoresis was performed according to the PulseNet standardized protocol for *V. cholerae* (*14*). Gel Compare II software (Applied Maths NV, Sint-Martens-Latem, Belgium) was used for electrophoresis pattern comparison that runs on Dice similarity index and unweighted pairgroup with arithmetic mean method.

Among the 400 isolates presumptively identified NAG vibrios, multiplex PCR confirmed 131 and 269 strains (each strain representing a case) as *V. fluvialis* and *V. cholerae*, respectively. The overall prevalence rate of *V. fluvialis* among 11,904 hospitalized patients with diarrhea was 1.1%. Abrupt appearance of *V. fluvialis* was identified in 2002, although the surveillance of diarrheal infection was initiated at the Infectious Diseases and Beliaghata General Hospital in 1996 (www.niced.org.in/annual_reports.htm). The isolation rate of *V. fluvialis* gradually increased from 0.7% in 2002 to 2.2% in 2009 (Table 1). Of the 131 strains of *V. fluvialis*, 43 (33%) were identified as the sole pathogen; the remaining 88 (67%) were isolated as a co-pathogen with either *V. cholerae*, *V. parahaemolyticus*, *E. coli*, *Shigella* spp., parasites, or enteric viruses (data not shown). Among the mixed infections, *V. fluvialis* with *V. cholerae* was isolated most often (17%), followed by *V. fluvialis* and *V. parahaemolyticus* (8%). The presence of *Vibrio* spp. as mixed pathogens indicates that these patients likely acquired the infection from contaminated water or food. We analyzed the date of admission and place from where the patients resided and found no evidence for clusters of infection or small outbreaks caused by *V. fluvialis.*

**Table 1:** Prevalence of *Vibrio fluvialis* among patients with diarrhea, Kolkata, India, 2002–2009

Year	No. samples	No. (%) *V. fluvialis* isolates	No. (%) patients	
			Sole infection	Mixed infection
2002	2,285	16 (0.7)	5 (0.2)	11 (0.5)
2003	1,673	8 (0.5)	1 (0.1)	7 (0.4)
2004	2,430	19 (0.8)	6 (0.2)	13 (0.5)
2005	1,472	17 (1.1)	7 (0.5)	10 (0.7)
2006	930	12 (1.3)	4 (0.4)	8 (0.9)
2007	842	9 (1.1)	2 (0.2)	7 (0.8)
2008	1,124	24 (2.1)	8 (0.7)	16 (1.4)
2009	1,153	26 (2.2)	10 (0.9)	16 (1.4)
Total	11,909	131 (1.1)	43 (0.4)	88 (0.7)

*V. fluvialis* infection was much more often detected in adults (73%) than in children <5 years of age (27%). Clinical symptoms of sole infection caused by *V. fluvialis* were similar to that of cholera: watery diarrhea (86%), severe dehydration status (28%), and abdominal pain (12%) (Table 2). Several previous investigations have identified cholera-like diarrheal outbreaks caused by *V. fluvialis* (*4*,*5*).

**Table 2:** Clinical features of *Vibrio fluvialis*–infected patients with diarrhea, Kolkata, India, 2002–2009

Clinical feature	No. (%) patients	
	Sole infection	Mixed infection
Type of diarrhea		
Watery	36 (86)	72 (81)
Bloody mucus, loose	7 (16)	16 (19)
Dehydration status		
Severe	12 (28)	14 (16)
Some or rare	31 (72)	74 (84)
Fever		
Yes	4 (9)	9 (10)
No	39 (91)	79 (90)
Abdominal pain		
Yes	5 (12)	11 (12)
No	38 (88)	77 (88)
Age		
>5 y	30 (70)	66 (75)
<5 y	13 (30)	22 (25)
Sex		
M	23 (53)	58 (66)
F	20 (47)	30 (34)

All the *V. fluvialis* strains were negative for the virulence genes commonly reported in *V. cholerae* and *V. parahaemolyticus*, but >90% were positive for genes encoding VFH and metalloproteases. More than 80% of the strains expressed hemolysin against rabbit and sheep red blood cells. Hemolysin is a widely distributed virulence factor in most pathogenic *Vibrio* spp. Metalloprotease produced by *V. fluvialis* is related to hemagglutination proteases of *V. vulnificus*, which enhances permeability and hemorrhagic activities (*12*). These factors may increase the virulence of *V. fluvialis* and contribute to diarrhea.

When the culture filtrates were tested, cytotoxic effect was readily noticed in the Chinese hamster ovary and HeLa cell lines, i.e., cytoplasmic vacuolation, cell rounding, and destruction of the monolayer. In most strains isolated as a sole pathogen, the cytotoxic endpoint titer was 2–256 (Technical Appendix Table 1). The cell vacuolation phenomenon has been reported as a virulence factor in several enteric pathogens (Technical Appendix References).

In this study, *V. fluvialis* strains were highly resistant to ampicillin (92%), streptomycin (85%), furazolidone (85%), and sulfamethoxazole/trimethoprim (70%) (Technical Appendix Table 2). About half the number of strains were resistant to ciprofloxacin and 45% to nalidixic acid; the lower resistance rate for nalidixic acid compared with fluoroquinolones is unexpected and warrants further investigation to confirm the additional mechanisms. In a previous study, we found that some *V. fluvialis* strains carried the plasmid-mediated quinolone resistance gene allele *qnrA1* and a gene encoding the aminoglycoside acetyltransferase (*aac(6')-Ib-cr*), which reduces ciprofloxacin activity (*15*). Fluroquinolone resistance and intermediate susceptibility to erythromycin (92%) are the unique features of the *V. fluvialis* isolated in this study; this trend was not recorded in other *Vibrio* spp., e.g., *V. cholerae* and *V. parahaemolyticus*.

Although the *V. fluvialis* strains exhibited distinct *Not*I restriction profiles in the denrogroam analysis, at least 4 major clades were identified (Figure). Clades A and B, with strains isolated during 2002–2007, exhibited less antimicrobial drug resistance than did clade C and D strains identified during 2008–2009; multidrug-resistant strains, especially those resistant to fluroquinolones, were identified in higher numbers in clades C and D (Figure).

**Figure:**
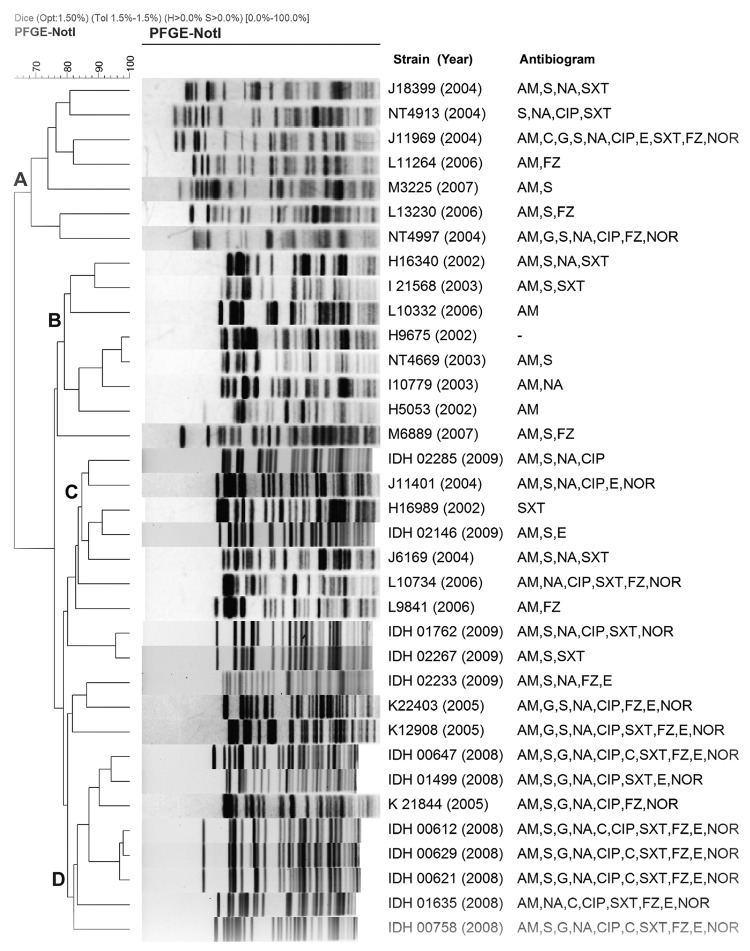
Dendrogram of *Not*I-digested pulsed-field gel electrophoresis (PFGE) profiles with representative *Vibrio fluvialis* isolates. Clustering identified 4 clades (A–D). AM, ampicillin; S, streptomycin; G, gentamicin; NA, nalidixic acid; CIP, ciprofloxacin; C, chloramphenicol; E, erythromycin; SXT, sulfamethoxazole-trimethoprim; FZ, furazolidone; NOR, norfloxacin. Scale bar indicates degree of similarity.

## Conclusions

Our results demonstrate an emerging trend of prevalence of *V. fluvialis* among patients with acute diarrhea patients in Kolkata. The expression of cytotoxic activity and hemolysin may contribute to understanding the pathogenicity of *V. fluvialis*. Further epidemiologic studies are necessary to elucidate the public health importance of *V. fluvialis*–mediated diarrhea.

Technical Appendix Results of assays of clinical *Vibrio fluvialis* strains to determine ability to lyse rabbit erythrocytes and cytotoxic effect on Chinese hamster ovary and HeLa cells and antimicrobial drug resistance of *V. fluvialis.*
