# Supplementary material for: Vibrio fluvialis in Patients with Diarrhea, Kolkata, India
Source: Emerg Infect Dis. 2012 Nov;18(11):1868–71. doi: 10.3201/eid1811.120520 (PMC3559161; doi:10.3201/eid1811.120520)
Supplement: Technical Appendix — Results of assays of clinical Vibrio fluvialis strains to determine ability to lyse rabbit erythrocytes and cytotoxic effect on Chinese hamster ovary and HeLa cells and antimicrobial drug resistance of V. fluvialis. [file 12-0520-Techapp-s1.pdf]

Article DOI: http://dx.doi.org/10.3201/eid1811.120520

Vibrio fluvialis in Patients with Diarrhea, Kolkata, India

Technical Appendix

Technical Appendix Table 1. Results of assays of clinical *Vibrio fluvialis* strains to determine ability to lyse rabbit erythrocytes and cytotoxic effect on CHO and HeLa cells.

				Cytotoxic titer	
Strain identification	*V. fluvialis* hemolysin	*V. fluvialis* metalloprotease	Hemolytic titer	Chinese hamster ovary	HeLa
IDH00612	+	+	16	64	4
IDH00629	+	+	2	4	2
IDH00653	+	+	4	32	16
IDH01036	+	+	32	32	8
IDH01577	+	+	4	16	16
H8942	+	+	8	16	8
H17768	+	+	16	8	4
L15318	+	+	2	2	2
K24681	+	+	64	128	256
J11969	+	+	32	32	128

Technical Appendix Table 2. Antimicrobial drug resistance of *Vibrio fluvialis*

Drug	Resistant, %	Intermediate, %
Ampicillin	92	8
Chloramphenicol	15	45
Ciprofloxacin	50	20
Sulfamethoxazole/ trimethoprim	70	14
Erythromycin	8	92
Furazolidone	85	15
Gentamicin	22	10
Nalidixic acid	45	15
Neomycin	10	88
Norfloxacin	40	35
Streptomycin	85	15
Tetracycline	10	15

References

1. Figueroa-Arredondo P, Heuser JE, Akopyants NS, Morisaki JH, Giono-Cerezo S, Enríquez-Rincón F, et al. Cell vacuolation caused by *Vibrio cholerae* hemolysin. Infect Immun. 2001; 69:1613–24.

2. Abrami L, Fivaz M, Glauser PE, Parton R, Van der Goot FG. A pore-forming toxin interacts with a GP-I-anchored protein and causes vacuolization of the endoplasmic reticulum. J. Cell Biol. 1998; 140:525–40.

3. Istivan TS, Coloe PJ, Fry BN, Ward P, Smith SC. Characterization of a haemolytic phospholipase A(2) activity in clinical isolates of *Campylobacter concisus*. J Med Microbiol. 2004;53:483–93.
